# Harnessing Artificial Intelligence for Enhanced Renal Analysis: Automated Detection of Hydronephrosis and Precise Kidney Segmentation

**DOI:** 10.1016/j.euros.2024.01.017

**Published:** 2024-02-22

**Authors:** Radu Alexa, Jennifer Kranz, Rafael Kramann, Christoph Kuppe, Ritabrata Sanyal, Sikander Hayat, Luis Felipe Casas Murillo, Turkan Hajili, Marco Hoffmann, Matthias Saar

**Affiliations:** a Department of Urology and Pediatric Urology, University Hospital, RWTH Aachen University, Aachen, Germany; b Department of Urology and Kidney Transplantation, Martin Luther University, Halle (Saale), Germany; c Department of Nephrology, Rheumatology, Clinical Immunology and Hypertension, RWTH Aachen, Aachen, Germany; d Computer Science, University of Texas at Dallas, USA; e Robotic Systems Engineering, RWTH Aachen University, Aachen, Germany

**Keywords:** Hydronephrosis, Artificial intelligence, Segmentation, Neuronal networks

## Abstract

**Background and objective:**

Hydronephrosis is essential in the diagnosis of renal colic. We automated the detection of hydronephrosis from ultrasound images to standardize the therapy and reduce the misdiagnosis of renal colic.

**Methods:**

Anonymously collected ultrasound images of human kidneys, both normal and hydronephrotic, were preprocessed for neural networks. Six “state of the art” models were trained and cross-validated for the detection of hydronephrosis, and two convolutional networks were used for kidney segmentation. In the testing phase, performance metrics included true positives, true negatives, false positives, false negatives, accuracy, and F1 score, while the evaluation of the segmentation task involved accuracy, precision, dice, jaccard, recall, and ASSD.

**Key findings and limitations:**

A total of 523 sonographic kidney images (423 nonhydronephrotic and 100 hydronephrotic) were collected from three different ultrasound devices. After training on this dataset, all models were used to evaluate 200 new ultrasound kidney images (142 nonhydronephrotic and 58 hydronephrotic kidneys). The highest validation accuracy (98.5%) was achieved by the AlexNet model (GoogLeNet 97%, AlexNet_v2 96%, ResNet50 96%, ResNet101 97.5%, and ResNet152 95%). The deeplabv3_resnet50 and deeplabv3_resnet101 reached a dice coefficient of 94.74% and 94.48%, respectively, on the task of automated kidney segmentation. The study is limited by analyzing only hydronephrosis, but this specific focus enabled high detection accuracy.

**Conclusions and clinical implications:**

We show that our automated ultrasound deep learning model can be trained and used to interpret and segmentate ultrasound images from different sources with high accuracy. This method will serve as an automated tool in the diagnostic algorithm of acute renal failure in the future.

**Patient summary:**

Hydronephrosis is crucial in the diagnosis of renal colic. Recent advances in artificial intelligence allow automated detection of hydronephrosis in ultrasound images with high accuracy. These methods will help standardize the diagnosis and treatment renal colic.

## Take Home Message

Recent advances in artificial intelligence allow automated detection of hydronephrosis in ultrasound images with high accuracy. These methods pave the way for automated standardized diagnosis and treatment of patients with renal colic.

## 1 Introduction

Hydronephrosis is a major criterion in diagnosing acute renal colic [1]. It indicates the possible presence of obstruction (eg, urolithiasis) or reflux in the upper urinary tract, and shows up on ultrasound as a hypoechogenic fluid in both the renal pelvis and the calyces. With 750 000 cases per year in Germany, urolithiasis significantly impacts the population's quality of life and socioeconomic factors [2]. According to Smith-Bindman et al [1], utilizing ultrasonography as the initial diagnostic tool in suspected nephrolithiasis cases has been shown to reduce the necessity for computed tomography scans, resulting in lower radiation exposure and no increase in serious adverse events, pain, or hospital visits. A substantial variation in the treatment of renal colic has also been documented. Imaging and blood tests are performed in about half of patients, urinalysis is not performed in one-fifth of patients, and antibiotics are incorrectly prescribed to one-fourth of patients [3]. Based on these data, a standardized and automated initial triage of patients, incorporating various clinical and hematological information, including the potential identification of hydronephrosis through ultrasound evaluations of the kidneys, has the potential to mitigate radiation exposure, improve diagnostic accuracy, and enhance subsequent treatment of renal colic. Recent advances in deep learning neural networks (NNs), imaging techniques, and computational capabilities have facilitated image-based pattern recognition within different datasets. These models have already been used for predictive pattern discovery [4], [5] and in the image-based detection of various urological pathologies [6], [7].

Using an ultrasound database of urological organs, an NN can successfully be trained to distinguish the kidneys from other urological organs and automatically detect the presence of hydronephrosis.

In cases of renal colic, our clinical experience suggests that the presence of kidney dilation, in conjunction with other clinical and laboratory features, plays a pivotal role in diagnostic and therapeutic decision-making processes, placing more emphasis on its existence rather than its extent.

As part of our endeavor to develop software that aids in triage and offers automated therapeutic recommendations for patients with renal colic in the emergency room before contact with doctors, we have created an NN designed to detect kidney dilation automatically.

## 2 Patients and methods

**Fig. 1:**
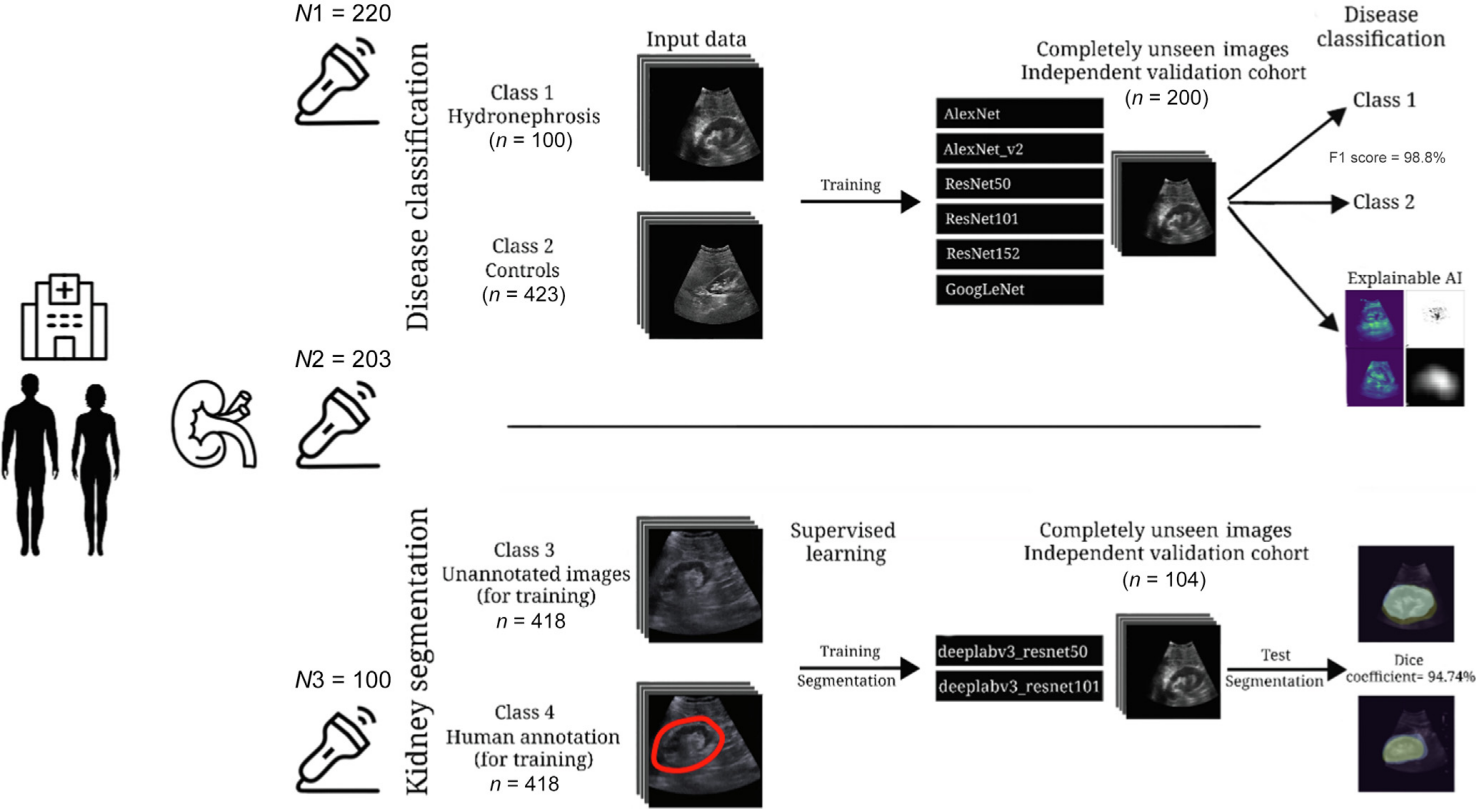
Comprehensive visual summary depicting the paper workflow structure: real ultrasound kidney images were collected with three different ultrasound devices (N1, N2, and N3) from patients with hydronephrosis (class 1) and no hydronephrosis (class 2), and used to train six neuronal networks (AlexNet, AlexNet_v2, ResNet50, ResNet101, ResNet152, and GoogLeNet) for disease classification (hydronephrosis or no hydronephrosis) and two neuronal networks (deeplabv3_resnet50 and deeplabv3_resnet101) for the segmentation task. Afterward, the neural networks were tested on unseen images, with the best F1 score in the disease classification reached by AlexNet and the best dice coefficient in the segmentation task by deeplabv3_resnet50. Using specific filters on the AlexNet, it was shown on which part of the image the neural network focuses on disease classification. AI = artificial intelligence.

We created a local image database of anonymized ultrasound images from three different ultrasound devices from our department (Fig. 1).

### 2.1 Image preprocessing and NNs

Image preprocessing and image augmentation are important steps in machine learning, as it helps standardize images and reduce noise [8]. This step also improves the ability of models to learn relevant information from the database [9]. To simulate the conditions of the ultrasound examination in which the probe can take different rotation positions, we applied random image augmentation to our dataset and rotate the images. Moreover, the contrast of the ultrasound images is affected by the imbalanced distribution of the ultrasound waves by the position of the kidney on different depths or by the interposition of different structures between the probe and the organ (ie, adipose tissue, liver, spleen, etc.). Accordingly, to simulate reality, we further augmented the dataset with different degrees of brightness.

The image preprocessing and NN training were done using the python Pytorch library [10]. The images were first cropped, resized (224 × 224 pixels), and normalized. Subsequently, data augmentation was performed using Pytorch random augmentations; the method is based on the RandAugment method proposed by Cubuk et al [11]. The augmentations included random horizontal flips, rotations with a random angle, and random applications of filters with a default probability of 50%. For image classification, we opted to use NN architectures present within the state of the art (SotA) algorithms [12], [13], [14]. Consequently, the NNs created were based on the ResNet [12], AlexNet [13], and GoogLeNet [14] architectures. Some minor changes were made to the architectures and their parameters before training them with our dataset. First, the AlexNet architecture was implemented with 0.5 dropout layers and the ReLU activation functions. Furthermore, the input was adapted for grayscale images by reducing the input channels to 1 instead of 3 normally used for RGB images. Second, the GoogLeNet architecture was also adapted for grayscale images, and dropouts of 0.2 and 0.7 were used for the main and auxiliary output layers, respectively. Finally, the ResNet architectures used within this work were based on the implementations offered by the Pytorch torchvision package [15] adapted for grayscale images. Every NN was trained using the cross-entropy loss and the Adam Optimizer with a learning rate of LR and weight decay of 0.0005.

As metrics for evaluation, the F1 score and the accuracy of the trained NN models were calculated within an unseen data test set. Both the accuracy and the F1 score are metrics commonly used within machine learning to assess model performance. These are functions of the true negatives (TNs), true positives (TPs), false negatives (FNs), and false positives (FPs) of the test data samples. The accuracy is the number of correctly labeled data over the total number of data samples, while the F1 score is calculated as shown in Supplementary Figure 1.

### 2.2 Automated kidney segmentation

For the segmentation models, each image data sample was accompanied by its corresponding mask represented by a red contour surrounding the area of interest, that is, the kidney. Therefore, the masks were extracted and transformed into binary images within the preprocessing of the images. Subsequently, the images and their masks were cropped, resized, and normalized as in the classification datasets. Furthermore, random data augmentation, similar to the approach used in the hydronephrosis detection dataset, was applied to the segmentation data samples [11].

The Deeplabv3 models [10] were chosen from the SotA to perform semantic segmentation on our dataset, specifically the ResNet50 and ResNet101 architectures. During implementation, we found that the pretrained models available within the torchvision [10] package drastically outperformed the models trained from scratch. Consequently, the images were transformed to RGB to be compatible with the pretrained models. Our models were initialized using the pretrained ones as a starting point and further trained with our dataset. To assess the model training performance, the image pixel-wise precision and accuracy were calculated. Each pixel of the output image was categorized as TP, TN, FN, or FP with respect to the original mask. Then, for the validation, we calculated the accuracy, precision, dice, jaccard, recall, and ASSD score of the segmentation networks on 50% of the test dataset.

**Fig. 2:**
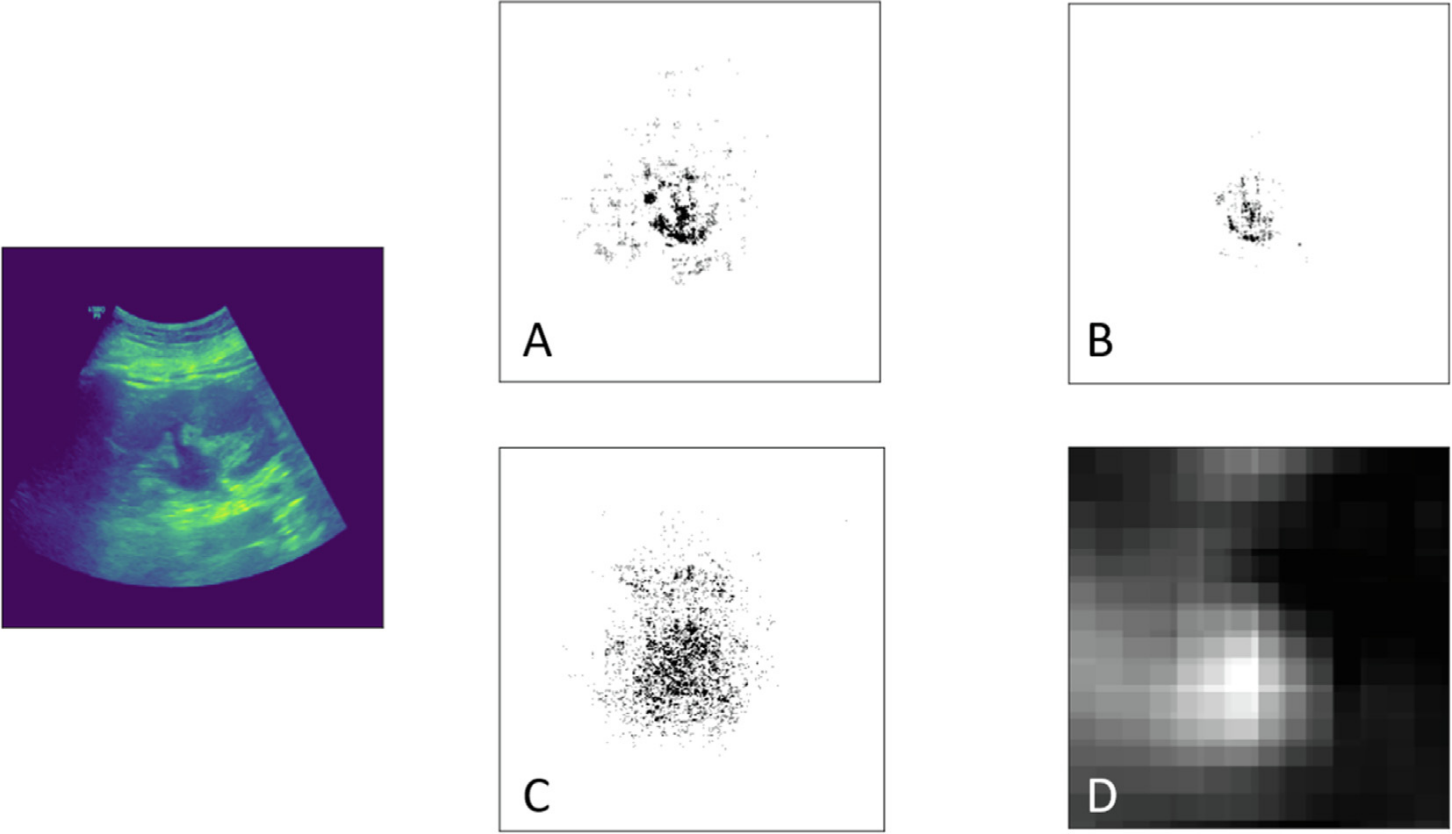
Example of all filters applied to one ultrasound image for the explainable artificial intelligence with the AlexNet model: (A) integrated gradients, (B) noise tunnel attribution, (C) gradient SHAP, and (D) occlusion based attribution (for Fig. 2A–C, the focus of the network is shown in black—more intense, more relevant the area in the diagnosis of the hydronephrosis; for Fig. 2D, the focus of the network is shown in white—more intense, more relevant the area in the diagnosis of the hydronephrosis). SHAP = Shapley Additive Explanations.

Explainable artificial intelligence (XAI) algorithms were used to analyze the output of our models. To this end, we used the Captum python library [16] to implement the XAI algorithms. First, the outputs were analyzed using the integrated gradient method proposed by Sundararajan et al [17]. It is a representation of the integral of gradients with respect to inputs along a dimension of the input. Next, the gradient Shapley Additive Explanations (SHAP) were calculated. Gradient SHAP calculates the expected values of gradients at random points between a baseline and the input with Gaussian noise [18]. Furthermore, the noise tunnel attribution method was performed in the data samples, where the attribution is computed multiple times while incrementally adding Gaussian noise to the sample (Fig. 2) [19]. Finally, an occlusion-based method was used where contiguous patches of the image are replaced with the baseline and the output difference is computed. The resulting images represent a heatmap of the most significant patches of the original data sample [20].

### 2.3 Software and hardware

Our analyses were based on Python 3.10 (Python Software Foundation, Wilmington, DE, USA) and were built in Pytorch. All analyses were performed on a computer with 2 Intel Xeon Gold processor 6248R (Intel, Santa Clara, CA, USA) and an Santa Clara, California, USA NVIDIA GeForce RTX 3090 with 24 GB RAM.

### 2.4 Ultrasound devices

The ultrasound equipment employed to acquire the images comprised Herlev, Denmark BK with the 5000 (N1) and Specto (N2) models and Solingen, Nordrhein-Westfalen, Deutschland GE using Logiq P9 (N3). The images were all two dimensional and were captured using B-mode ultrasound with the help of convex abdominal probes (2–5 MHz) while the patients were lying on their back.

### 2.5 Ethics

This study followed German data regulations and the Declaration of Helsinki, and was approved by our local ethical committee (EK 22-360).

## 3 Results

A total of 723 ultrasound images were collected from three different ultrasound devices (see Table 1). Of the images, 523 were used as the training set and 200 as the test set. The most images from the training set were recorded with the BK 5000 device (N1) followed by BK Specto (N2) and GE Logiq P9 (N3).

**Table 1:** Data set: device 1 (N1—BK 5000), device 2 (N2—BK Specto), and device 3 (N3—GE Logiq P9)

	N1, *n* (%)	N2, *n* (%)	N3, *n* (%)	Total
*Training set*				
Normal	191 (36.52)	176 (33.65)	56 (10.71)	423
Hydronephrosis	29 (5.55)	27 (5.16)	44 (8.41)	100
Total	220 (42.07)	203 (38.81)	100 (19.12)	523
*Test set*				
Normal	68 (34)	39 (19.50)	35 (17.50)	142
Hydronephrosis	14 (7)	15 (7.50)	29 (14.50)	58
Total	82 (41)	54 (27)	64 (32)	200

N = number of images per device.

All six models were trained in order to achieve the best F1 score. All the models were trained using the Adam optimizer for 1500 epochs, whereas GoogLeNet was trained for 750 epochs. GoogLeNet achieved the highest training accuracy of 100%, while ResNet50 and ResNet152 obtained the highest validation accuracy of 98.07%. Overall, the models exhibited high training accuracies, but varied in validation accuracies and training durations. These training characteristics are presented in Supplementary Table 1.

In the test phase, we analyzed the performance metrics of our six deep learning models in terms of test accuracy, recall, precision, F1 score, and the difference in F1 score compared with the AlexNet. AlexNet exhibits the highest F1 score of 98.95%, test accuracy of 99.52%, recall of 99.3%, and a precision of 98.6%, setting the baseline for F1 score difference as 0. AlexNet_v2 and ResNet152 have deviations in F1 score from AlexNet, with 1.71% and 2.35%, respectively. ResNet101 achieved the closest F1 score to AlexNet with only a 0.7% difference. All models showcased high performance, with test accuracies ranging from 96.15% to 99.52%, and ResNet152 achieved a recall of 100%. The confusion matrix indicating the evaluation of all models is presented in Table 2.

**Table 2:** Confusion matrix for the neural networks trained on the data set

	Test accuracy (%)	Recall (%)	Precision (%)	F1 score (%)	Difference F1 score (AlexNet – actual), (%)
AlexNet	99.52	99.3	98.6	98.95	0
AlexNet_v2	96.15	99.3	95.27	97.24	1.71
ResNet50	98.07	97.89	96.53	97.2	1.75
ResNet101	96.15	98.59	97.9	98.25	0.7
ResNet152	98.07	100	93.42	96.6	2.35
GoogLeNet	98.07	96.48	99.28	97.86	1.09

**Fig. 3:**
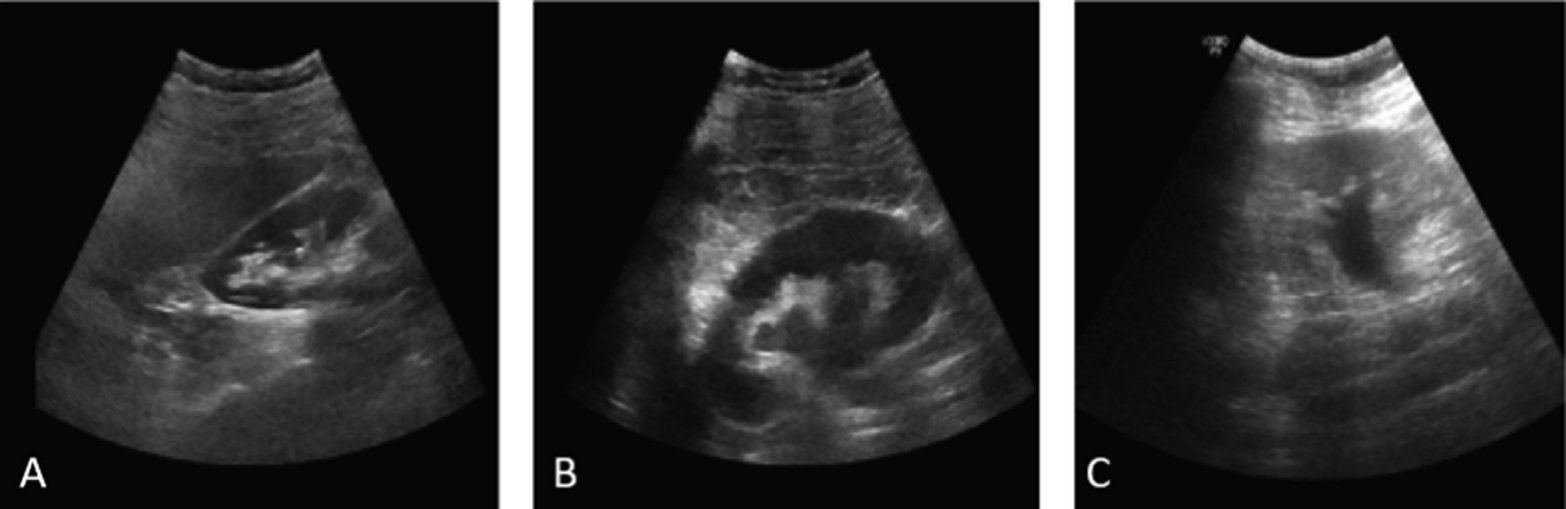
Misinterpreted images from AlexNet: (A) normal kidney misinterpreted as hydronephrotic; (B and C) hydronephrotic kidneys misinterpreted as normal.

Three images were misclassified by the AlexNet (one image with no hydronephrosis as hydronephrosis and two images with hydronephrosis as no hydronephrosis; Fig. 3). Figure 3A shows a hypoechogenic structure in the middle of the renal calyx system, which could be misinterpreted as a dilated middle calyx. Figure 3B was misinterpreted as normal, although with a low probability (65%) of a correct diagnosis. Figure 3C had unclear margins of the kidney, which eventually mislead the diagnosis.

In order to understand and interpret the predictions of the models, we used four different types of filters (activation maps). The activation maps for the AlexNet model highlight the focus of the NN when it analyzes the image for hydronephrotic characteristics (Supplementary Fig. 2).

For the segmentation task, we trained two fully convolutional networks: deeplabv3_resnet50 and deeplabv3_resnet101. DeeplabV3 with ResNet50 has test accuracy of 90.21% and a dice score of 94.74. In comparison, DeeplabV3 with ResNet101 scores slightly better in test accuracy at 90.37%, has nearly identical precision of 99.28%, and a marginally lower dice score of 94.48. The confusion matrix of the NNs in the test phase is shown in Supplementary Table 2.

**Fig. 4:**
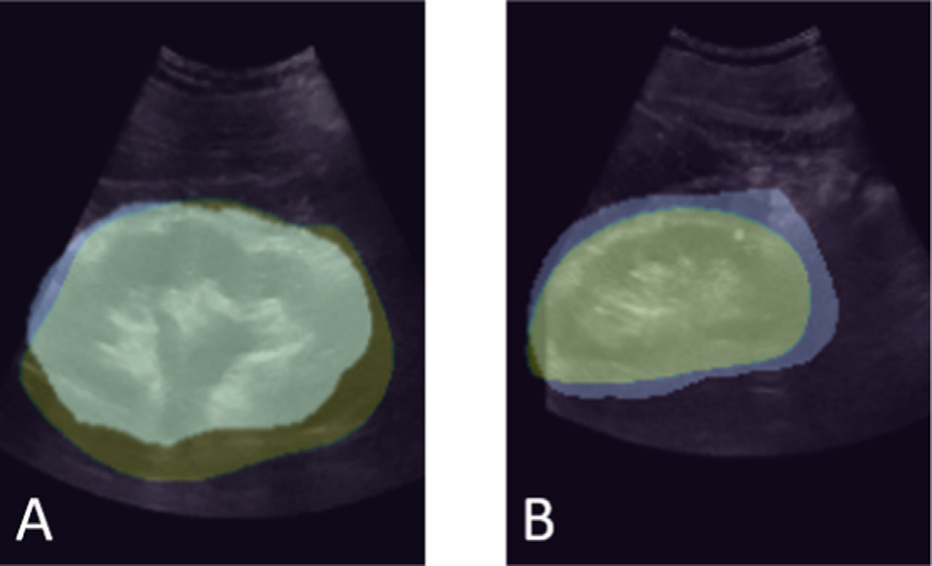
Manually and automated segmented kidney images: (A) kidney with hydronephrosis and (B) kidney without hydronephrosis. Yellow color indicates manually segmented, blue color indicates automated segmentation with the deeplabv3_resnet50—fully convolutional network, and overlapped image indicates both manual and automated segmentation employed.

Two examples of automated segmentation with the deeplabv3_50 are presented in Figure 4.

## 4 Discussion

In addition to utilizing deep learning techniques for predictive purposes, such as estimating the success rate of stone removal with shockwave lithotripsy and anticipating the need for readmission to the intensive care unit following ureteroscopy, artificial intelligence (AI) models have made significant advancements in risk stratification and the field of image recognition [4], [5], [6], [21], [22], [23], [24]. These models have demonstrated state-of-the-art performance in image recognition and segmentation due to their robust and accurate classification capabilities [25], [26]. The application of deep learning for image segmentation in computer or magnetic resonance imaging has already been reported for the prostate, bladder, kidney, and urolithiasis [27], [28], [29], [30].

This study illustrates the feasibility of employing AI for the recognition of ultrasound images depicting kidneys with hydronephrosis. Smail et al [7] evaluated the efficacy of a convolutional neuronal network model with five convolutional layers, trained to categorize prenatal hydronephrosis according to the 5-point Society for Fetal Urology classification system. This model achieved overall accuracy of 94%. Lien et al [31] adapted three deep learning models (U-Net, Res-UNet, and UNet++) and assessed their accuracy in detecting hydronephrosis through ultrasound images. The most effective model (Res-UNet) reached an accuracy of 94.6%.

Our AlexNet model accurately classified ultrasound images as not hydronephrotic or hydronephrotic, with overall accuracy of 98.5%. To our knowledge, it is the highest accuracy of an NN in the detection of hydronephrosis. The ability of the AI model to rapidly analyze large volumes of imaging data can save time, improve the efficiency of the diagnostic process, and reduce interobserver variability in the interpretation of ultrasound images.

Nonetheless, the use of AI raises concerns about the transparency and accountability of the decision-making process. Therefore, this model integrated elements of XAI to determine which features from the image contribute most to the NN output (Supplementary Fig. 2). By presenting the suspicious area of the organ, we ensure the model's transparency and transfer the decision-making to trained medical personnel.

A further section of our analysis is the automated segmentation of the kidney in ultrasound images as a key part of computer-aided diagnosis systems. This feature represents the extraction of representative regions from the ultrasound image (in our case, kidneys) to better describe the target organ and improve diagnostics. Chen et al [32] developed a fully automatic kidney segmentation with a deep NN architecture, which segmentate the kidneys from different quality classes of images with a total accuracy of 98.8%. Here, we developed a pixel-wise segmentation model, which reached a dice coefficient of 94.7%. As the main purpose of our model was the detection of hydronephrosis, we reached sufficient accuracy to detect the kidneys and, subsequently, the specific characteristics of this pathology. Nonetheless, future work is needed to improve the total segmentation accuracy of our model.

The potential limitations of our study should be considered carefully, as these may affect the analysis performance of the models. First, we used still images and not a series of images of the whole organ, which may limit the diagnostic capabilities of the algorithm. Thus, the model's accuracy could be increased further by using a series of images. This study is exclusively focused on a single kidney pathology: hydronephrosis. The exclusion of images depicting various pathologies, such as cysts, venous dilations, large cavities in junctional syndromes, or tumors, along with the decision not to analyze all available ultrasound modes/images (B mode, M mode, Doppler, three-dimensional [3D] ultrasound data, cine clips, multimodal ultrasound images, and 3D images), may impact the hydronephrosis detection rate by introducing potential FPs or FNs. Nonetheless, in an end-to-end AI scenario for kidney analysis using ultrasound images, our model’s role becomes pivotal in hydronephrosis detection. Specifically designed to interpret hydronephrosis, our model stands out as the preferred choice, complementing other models that focus on different entities or pathologies. Another limitation is the number of images used to train the models. Although we have tried to consider all possible variations of the kidney locations, we could not consider all possible angles and degrees of brightness of the ultrasound findings. We aimed to overcome this limitation with in silico image augmentation.

## 5 Conclusions

In summary, our model reached high accuracy in differentiating between not hydronephrotic and hydronephrotic kidneys. After further training and improvements, this deep learning model could be integrated into AI-aided imaging diagnostic tools and also standardize the diagnosis and treatment of renal colic.

  ***Author contributions*:** Radu Alexa had full access to all the data in the study and takes responsibility for the integrity of the data and the accuracy of the data analysis.

  *Study concept and design*: Alexa, Saar.

*Acquisition of data*: Alexa.

*Analysis and interpretation of data*: Alexa, Casas, Hayat, Sanyal.

*Drafting of the manuscript*: Alexa.

*Critical revision of the manuscript for important intellectual content*: Saar, Kranz, Hoffmann, Hajili.

*Statistical analysis*: Alexa, Casas.

*Obtaining funding*: None.

*Administrative, technical, or material support*: Casas, Saar.

*Supervision*: Saar, Kranz, Kramann, Kuppe.

*Other*: None.

  ***Financial disclosures:*** Radu Alexa certifies that all conflicts of interest, including specific financial interests and relationships and affiliations relevant to the subject matter or materials discussed in the manuscript (eg, employment/affiliation, grants or funding, consultancies, honoraria, stock ownership or options, expert testimony, royalties, or patents filed, received, or pending), are the following: None.

  ***Funding/Support and role of the sponsor***: None.
