# Supplementary material for: Harnessing Artificial Intelligence for Enhanced Renal Analysis: Automated Detection of Hydronephrosis and Precise Kidney Segmentation
Source: Eur Urol Open Sci. 2024 Feb 22;62:19–25. doi: 10.1016/j.euros.2024.01.017 (PMC10998270; doi:10.1016/j.euros.2024.01.017)
Supplement: Supplementary data 1 [file mmc1.docx]

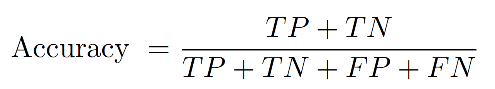


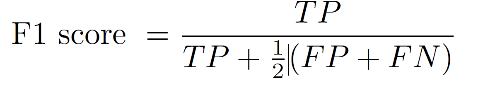


**Supplementary Figure 1.** Equation of accuracy and F1 Score

**Supplementary Table 1.** Characteristics of the training phase

|  | Training Accuracy | Validation Accuracy | Time for training | Epochs trained | Optimizer |
| --- | --- | --- | --- | --- | --- |
| AlexNet | 99,52 | 97,11 | 2h 49 min | 1500 | Adam |
| AlexNet_v2 | 99,28 | 96,15 | 2h 50 min | 1500 | Adam |
| ResNet50 | 99,76 | 98,07 | 3h 57 min | 1500 | Adam |
| ResNet101 | 99,76 | 96,15 | 5h 21 min | 1500 | Adam |
| ResNet152 | 99,52 | 98,07 | 6h 46 min | 1500 | Adam |
| GoogLeNet | 100 | 98,03 | 2h 2 min | 750 | Adam |


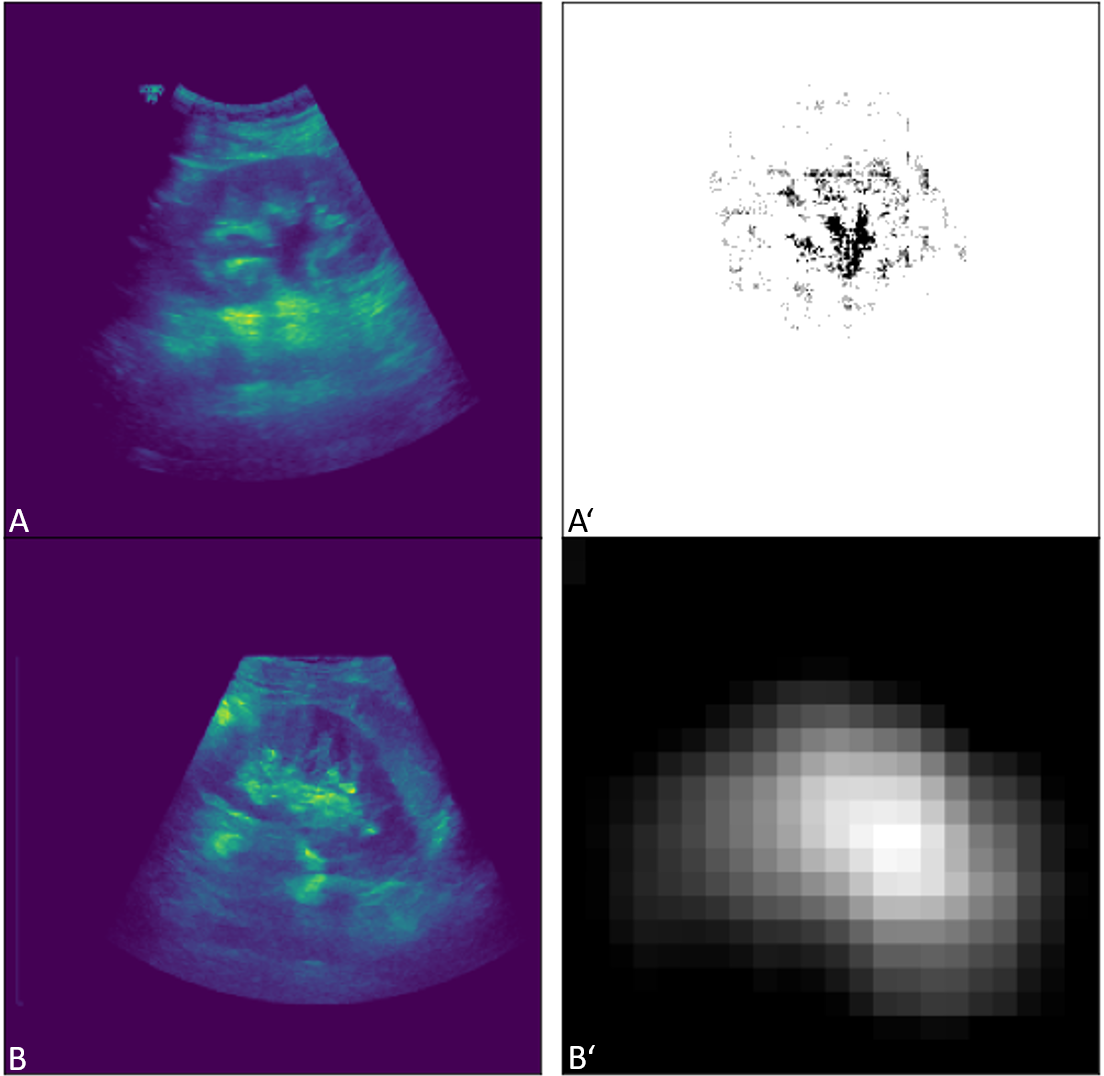


**Supplementary Figure 2.** Examples of the two classes and the applied explainable artificial intelligence (A – hydronephrosis, A’ - focus of the AlexNet through integrated gradient; B – normal kidney, B’ – focus of the AlexNet through Occlusion Based Attribution).

**Supplementary Table 2.** Confusion matrix for the segmentation neuronal networks

|  | Test Accuracy | Precision | Dice | Jaccard | Recall | ASSD |
| --- | --- | --- | --- | --- | --- | --- |
| deeplabv3_resnet50 | 90,21 | 99,29 | 94,74 | 90,08 | 90,74 | 6,37 |
| deeplabv3_reset101 | 90,37 | 99,28 | 94,48 | 90,25 | 91,93 | 6,3 |
